# Complementary Role of GlcNAc6ST2 and GlcNAc6ST3 in Synthesis of CL40-Reactive Sialylated and Sulfated Glycans in the Mouse Pleural Mesothelium

**DOI:** 10.3390/molecules27144543

**Published:** 2022-07-16

**Authors:** Yoshiko Takeda-Uchimura, Midori Ikezaki, Tomoya O. Akama, Kaho Nishioka, Yoshito Ihara, Fabrice Allain, Kazuchika Nishitsuji, Kenji Uchimura

**Affiliations:** 1 Unité de Glycobiologie Structurale et Fonctionnelle, UMR 8576 of the Centre National de la Recherche Scientifique, University of Lille, Villeneuve d’Ascq, F-59655 Lille, France; yoshiko.uchimura@univ-lille.fr (Y.T.-U.); fabrice.allain@univ-lille.fr (F.A.); 2 Department of Biochemistry, School of Medicine, Wakayama Medical University, Wakayama 641-8509, Japan; ikezaki@wakayama-med.ac.jp (M.I.); y-ihara@wakayama-med.ac.jp (Y.I.); nishit@wakayama-med.ac.jp (K.N.); 3 Department of Pharmacology, Kansai Medical University, Osaka 570-8506, Japan; akamat@hirakata.kmu.ac.jp; 4 Department of Obstetrics and Gynecology, School of Medicine, Wakayama Medical University, Wakayama 641-8509, Japan; ka-matsu@wakayama-med.ac.jp

**Keywords:** sulfotransferase, sulfated glycan, sialyl 6-sulfo Lewis X, sialomucin, mesothelium

## Abstract

Sialyl 6-sulfo Lewis X (6-sulfo sLe^X^) and its derivative sialyl 6-sulfo *N*-acetyllactosamine (LacNAc) are sialylated and sulfated glycans of sialomucins found in the high endothelial venules (HEVs) of secondary lymphoid organs. A component of 6-sulfo sLe^X^ present in the core 1-extended *O*-linked glycans detected by the MECA-79 antibody was previously shown to exist in the lymphoid aggregate vasculature and bronchial mucosa of allergic and asthmatic lungs. The components of 6-sulfo sLe^X^ in pulmonary tissues under physiological conditions remain to be analyzed. The CL40 antibody recognizes 6-sulfo sLe^X^ and sialyl 6-sulfo LacNAc in *O*-linked and *N*-linked glycans, with absolute requirements for both GlcNAc-6-sulfation and sialylation. Immunostaining of normal mouse lungs with CL40 was performed and analyzed. The contribution of GlcNAc-6-*O*-sulfotransferases (GlcNAc6STs) to the synthesis of the CL40 epitope in the lungs was also elucidated. Here, we show that the expression of the CL40 epitope was specifically detected in the mesothelin-positive mesothelium of the pulmonary pleura. Moreover, GlcNAc6ST2 (encoded by *Chst4*) and GlcNAc6ST3 (encoded by *Chst5*), but not GlcNAc6ST1 (encoded by *Chst2*) or GlcNAc6ST4 (encoded by *Chst7*), are required for the synthesis of CL40-positive glycans in the lung mesothelium. Furthermore, neither GlcNAc6ST2 nor GlcNAc6ST3 is sufficient for in vivo expression of the CL40 epitope in the lung mesothelium, as demonstrated by GlcNAc6ST1/3/4 triple-knock-out and GlcNAc6ST1/2/4 triple-knock-out mice. These results indicate that CL40-positive sialylated and sulfated glycans are abundant in the pleural mesothelium and are synthesized complementarily by GlcNAc6ST2 and GlcNAc6ST3, under physiological conditions in mice.

## 1. Introduction

Sialylated and sulfated glycans are enzymatically synthesized and are present in the *O*-linked and *N*-linked carbohydrate chains of various tissues. Sialyl 6-sulfo Lewis X (6-sulfo sLe^X^, Neu5Acα2-3Galß1-4(Fucα1-3)(6S)GlcNAc) is a major ligand for L-selectin that mediates immune-cell adhesion to the endothelium and promotes extravasation into secondary lymphoid tissues and sites of inflammation [1,2]. The 6-Sulfo sLe^X^ is abundant in the high endothelial venules (HEVs) of secondary lymphoid organs and regulates lymphocyte homing [3,4]. MECA-79 [5] is a well-known antibody that stains HEV and recognizes a component of 6-sulfo sLe^X^ present in core 1-extended *O*-glycans, requiring GlcNAc-6-sulfation but not α2,3 sialylation [1,6,7]. CL40 [8] and other anti-6-sulfo glycan antibodies [9,10,11] also recognize 6-sulfo sLe^X^ and stain HEV. Since GlcNAc-6-sulfation and α2,3 sialylation and dispensable α1,3 fucosylation occurs in its recognition mode, CL40 recognizes both 6-sulfo sLe^X^ and sialyl 6-sulfo N-acetyllactosamine (sialyl 6-sulfo LacNAc, Neu5Acα2-3Galß1-4(6S)GlcNAc) in *O*-linked and *N*-linked glycans [8]. It is known that MECA79- or CL40-reactive sulfated glycans are induced in a number of chronic pulmonary inflammation sites in animal models and patients. These include vasculature ligands expressed in allergic asthma, autoimmune diseases, viral infections, and lung allografts [12,13,14,15,16,17,18]. A component of 6-sulfo sLe^X^ is induced in lymphoid aggregate vasculature. Interestingly, this component is also found in luminal contents of the bronchus of asthmatic and allergic lungs [12]. However, 6-sulfo sLe^X^ and its derivatives remain to be further analyzed in normal lungs. Information about the presence and distribution of these sialylated and sulfated glycans within the lung in physiological conditions is useful in a comparative analysis with those in pathological conditions.

GlcNAc-6-sulfation is essential for CL40 recognition. GlcNAc-6-*O*-sulfotranferase (GlcNAc6ST) is a Golgi-resident enzyme that catalyzes the transfer of sulfate to the GlcNAc residue of 6-sulfo sLe^X^/sialyl 6-sulfo LacNAc. Five members of which are present in humans, and four of their orthologs in mice compose the GlcNAc6ST family [2]. We previously reported that GlcNAc6ST1 (encoded by the *Chst2* gene) [19] and GlcNAc6ST2 (encoded by the *Chst4* gene) [20,21] complement the synthesis of 6-sulfo sLe^X^ in HEV of lymph nodes [3,4,8]. The contribution of these GlcNAc6ST family members to other tissues is largely unknown. In the present study, we asked if 6-sulfo sLe^X^/sialyl 6-sulfo LacNAc physiologically occur in mouse lungs and investigated their histological localization using CL40. We also studied the contribution of GlcNAc6STs to the synthesis of the CL40 epitope by utilizing mice deficient in GlcNAc6ST1, GlcNAc6ST2, GlcNAc6ST3 (encoded by the *Chst5* gene), or GlcNAc6ST4 (encoded by the *Chst7* gene) [3,11,22,23,24]. We found that CL40-reactive sialylated and sulfated glycans are abundant in the pleural mesothelium of the normal lung. Unexpectedly, GlcNAc6ST2 and GlcNAc6ST3, but not GlcNAc6ST1 or GlcNAc6ST4, play complementary roles in the synthesis of mesothelial sialylated and sulfated glycans. Both GlcNAc6ST1,3,4 and GlcNAc6ST1,2,4 triple-deficient mice abolished CL40-reactive glycans in the mesothelium. These results indicate that 6-sulfo sLe^X^/sialyl 6-sulfo LacNAc selectively occurs in the pleural mesothelium of normal mouse lungs and is synthesized by GlcNAc6ST2 and GlcNAc6ST3.

## 2. Results and Discussion

### 2.1. CL40-Reactive Sialylated and Sulfated Glycans Are Abundant in the Mouse Pleural Mesothelium

**Figure 1:**
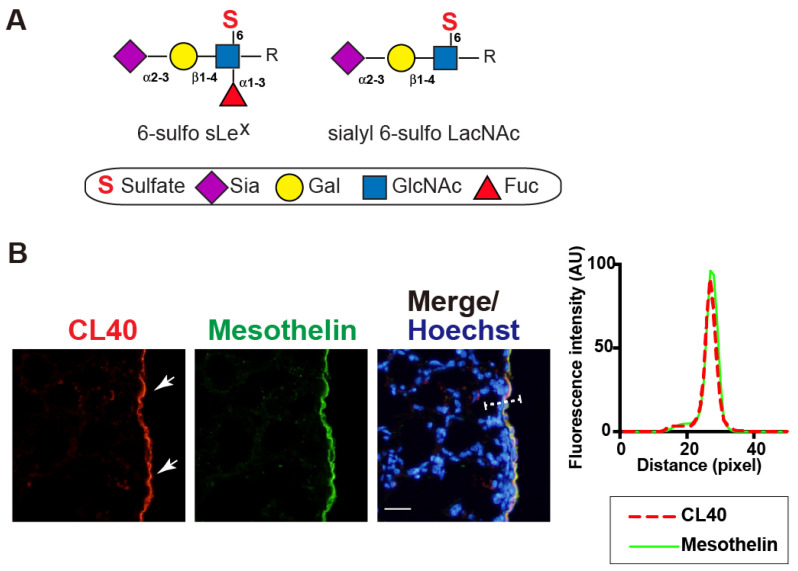
CL40-reactive sialylated sulfated glycans occur in the mouse pleural mesothelium. (**A**), Schematic representation of sialyl 6-sulfo Lewis X (left; 6-sulfo sLe^X^, Neu5Acα2-3Galß1-4(Fucα1-3)(6S)GlcNAc) and sialyl 6-sulfo *N*-acetyllactosamine (right; sialyl 6-sulfo LacNAc, Neu5Acα2-3Galß1-4(6S)GlcNAc). Both were recognized by CL40. C-6 sulfation (S), *N*-acetylneuraminic acid, predominant sialic acid (Sia), galactose (Gal), *N*-acetylglucosamine (GlcNAc), and fucose (Fuc) are shown. These glycans are extended from the variable underlying core glycans (R). sLe^X^ and mimetics thereof sulfated at C-6 of GlcNAc (“6-sulfo”) or C-6 of Gal (“6′-sulfo”) are present in mammals. (**B**) Lung sections from normal adult mice were co-stained with CL40 (red) and an anti-mesothelin antibody (green), followed by Hoechst 33342 nuclear staining (blue). Representative fluorescence microscopy images of the lower and middle regions of the left-lung lobe are shown (*n* = 3). Dense CL40 staining signals in the pleural mesothelium (arrows) revealed by co-staining with mesothelial marker mesothelin are shown. Digital images were captured using the same settings for each staining. The plot profile of CL40 and mesothelin staining is shown (right). The signal intensities along the path of the line marker (dashed white line) in the merged image were measured, as described in Materials and Methods. Scale bar: 20 µm.

CL40 antibody recognizes 6-sulfo sLe^X^ and sialyl 6-sulfo LacNAc [8] (Figure 1A). We investigated whether the sialylated and sulfated glycans were expressed in the mouse lung in a steady state. We found strong CL40 immunoreactivity in the pleural mesothelium (Figure 1B). These staining signals were co-localized with the staining signals of an antibody against mesothelin, a marker of mesothelial cells lining the lung pleura (Figure 1B). Immunostaining with an isotype-matched control (mouse IgG1) for CL40 gave no specific signals in the pleura. MECA79-staining signals were not observed, either (Figure S1). GlcNAc-containing fractions of mouse-lung lobes were prepared with wheat germ agglutinin (WGA)-coated beads. Western blot analysis for the bead-bound materials resulted in bands with sizes of >270 kDa and 145–175 kDa, which were immunoreactive for CL40 (Figure S2). Intensities of these bands were not reduced by PNGase F pretreatment (Figure S2). These results suggest that CL40-reactive glycans occur in high molecular-mass glycoproteins and that *O*-linked glycans modified with 6-sulfo sLe^X^ and/or sialyl 6-sulfo LacNAc are components of these glycoproteins that are densely present in the pleural mesothelium of adult mouse lungs.

**Figure 2:**
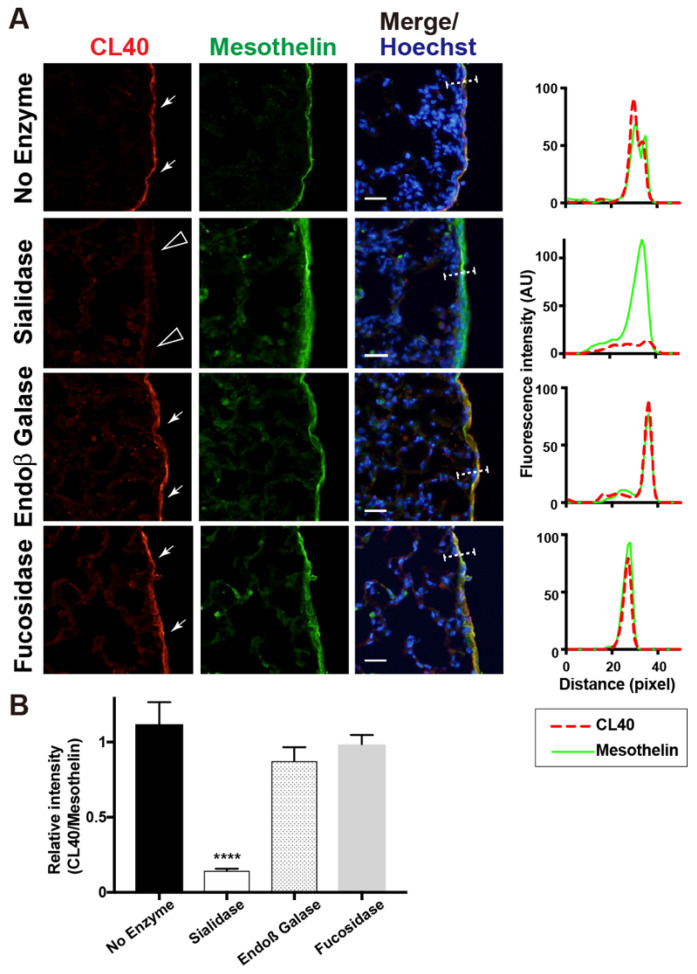
Sialidase pretreatment diminishes CL40 signals abundant in the mouse pleural mesothelium. (**A**) Lung sections from normal adult mice were co-stained with CL40 (red) and anti-mesothelin (green) followed by Hoechst 33342 nuclear staining (blue). Sections were pretreated with buffer only (no enzyme), α2-3,6,8 neuraminidase (sialidase), endo-ß-galactosidase (Endoß Galase), or α1-3,4 fucosidase (fucosidase). Representative fluorescence microscopy images of the lower/middle region of the left-lung lobe are shown (*n* = 3 for each treatment). Dense CL40 staining signals in the pleural mesothelium (arrows) revealed by co-staining with anti-mesothelin are shown. Digital images were captured using the same settings for each staining. Sections pretreated with α2-3,6,8 neuraminidase showed negligible levels of CL40 signals in the mesothelium (open arrowheads). The plot profiles of CL40 and mesothelin staining are shown (right). The signal intensities along the path of the line marker (dashed white line) in the merged images were measured, as described in Materials and Methods. (**B**) The relative intensity of CL40 to mesothelin is shown, with *n* = 12 mesothelium per treatment. Data are from two experiments, with 4 pleural mesothelium analyzed in lung specimens from three donors for each treatment. **** *p* < 0.0001. Scale bar: 20 µm.

We then investigated whether enzymatic removal of sialic acids could abolish CL40 immunoreactivity in the pleura. Lung sections were pretreated with α2-3,6,8 neuraminidase (sialidase). Sialidase-treated sections showed a negligible level of CL40 immunoreactivity, while the anti-mesothelin signals were retained (Figure 2). This is consistent with the fact that CL40 requires sialylation for its recognition [8], and that anti-mesothelin signals arose from the mesothelin core protein. Susceptibility to sialidase further supports the fact that CL40 recognized 6-sulfo sLe^X^/sialyl 6-sulfo LacNAc abundance in the mesothelium of the normal lung. We wanted to determine whether the CL40-reactive glycans were elongated from repeated GlcNAc-6-sulfated or non-sulfated LacNAc. Pretreatment of lung sections with endo-ß-galactosidase, an enzyme that cleaves GlcNAc-6-sulfated or non-sulfated poly-LacNAc [25], did not affect CL40 immunoreactivity (Figure 2). CL40-glycans may be rather short and composed of one LacNAc with sialylation and GlcNAc-6 sulfation. It has been suggested that the structure of CL40-reactive glycans is 6-sulfo sLe^X^ or sialyl 6-sulfo LacNAc, without additional LacNAc repeats on the side of the reducing end of the glycans. The α1-3,4 fucosidase pretreatment did not alter CL40 immunoreactivity (Figure 2). The major CL40-reactive glycan in the pleura may be sialyl 6-sulfo LacNAc, an afucosyl-type of 6-sulfo sLe^X^. Since sialic acids in close proximity to fucoses can inhibit efficient cleavage of glycans by fucosidase, further evaluation of fucosylation in CL40-reactive glycans may need to be performed.

### 2.2. GlcNAc6ST2 and GlcNAc6ST3 Are Required for the Synthesis of CL40-Reactive Sialylated and Sulfated Glycans in the Mouse Pleural Mesothelium

**Figure 3:**
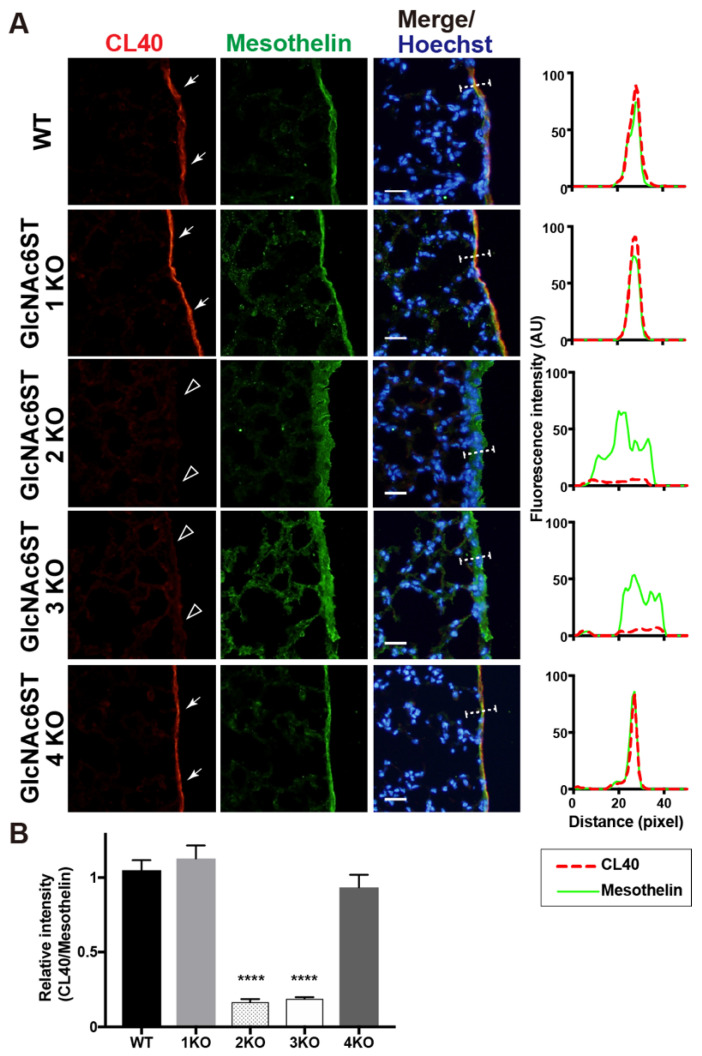
GlcNAc6ST2 and GlcNAc6ST3 are required for synthesis of CL40-reactive sialylated sulfated glycans in the mouse pleural mesothelium. (**A**) Lung sections prepared from normal wild-type (WT), *Chst2*-deficient (GlcNAc6ST1 KO) [3,22], *Chst4*-deficient (GlcNAc6ST2 KO) [3,26], *Chst5*-deficient (GlcNAc6ST3 KO) [23], or *Chst7*-deficeint (GlcNAc6ST4 KO) mice [24] were co-stained with CL40 (red) and anti-mesothelin (green) followed by Hoechst 33342 nuclear staining (blue). Dense CL40 staining in the pleural mesothelium (arrows) is shown. Sections of GlcNAc6ST2 KO and GlcNAc6ST3 KO mice showed negligible levels of CL40 signals in the mesothelium (open arrowheads). Digital images were captured using the same settings for each staining. The plot profiles of CL40 and mesothelin staining are shown (right). The signal intensities along the path of the line marker (dashed white line) in the merged images were measured, as described in Materials and Methods. *n* = 3 for each genotype. (**B**) The relative intensity of CL40 to mesothelin is shown, with *n* = 12 mesothelium per genotype. Data are from three experiments, with 4 pleural mesothelium analyzed in lung specimens from three donors for each genotype. **** *p* < 0.0001. Scale bar: 20 µm.

We previously showed that 6-sulfo sLe^X^ present in the HEV cells of peripheral lymph nodes is complementary to GlcNAc6ST1 and GlcNAc6ST2 [3,8]. We wanted to determine which GlcNAc6ST is responsible for the synthesis of CL40-reactive glycans in the mouse pleural mesothelium. Mice genetically deficient in each GlcNAc6ST gene were used for analysis. We first anticipated that one of the four GlcNAc6STs would be responsible for this. Unexpectedly, mice deficient in GlcNAc6ST2 or GlcNAc6ST3 showed trace or negligible levels of pleural CL40 immunoreactivity, whereas mice deficient in GlcNAc6ST1 or GlcNAc6ST4 showed levels comparable to those of WT mice (Figure 3). It is plausible that GlcNAc6ST2 and GlcNAc6ST3 complement mediate sulfation of CL40-reactive glycans in the pleural mesothelium in vivo.

**Figure 4:**
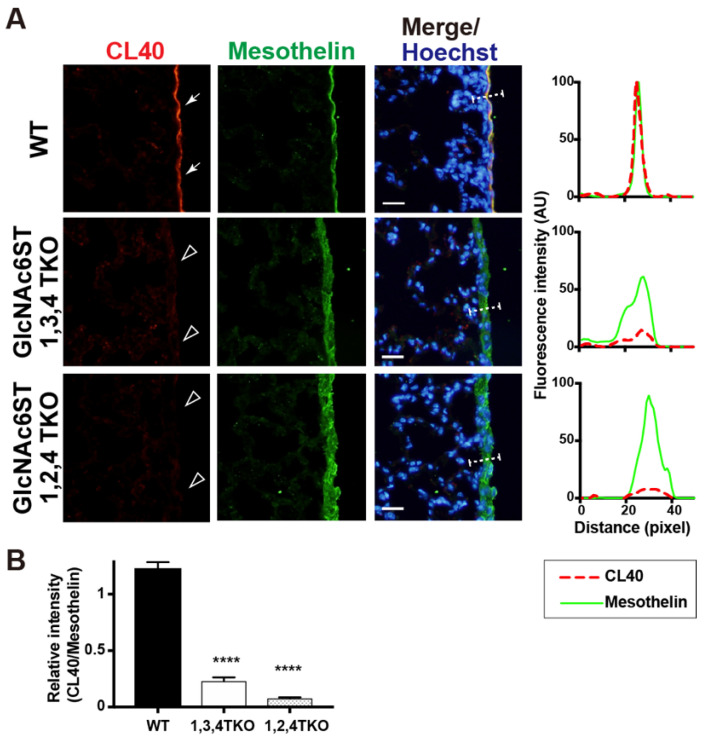
CL40-reactive sialylated sulfated glycans in the mouse pleural mesothelium are deficient in GlcNAc6ST1,3,4 triple KO and GlcNAc6ST1,2,4 triple KO mice. (**A**) Lung sections prepared from normal wild-type (WT), *Chst2/Chst5/Chst7* triple-deficient (GlcNAc6ST1,3,4 TKO), and *Chst2/Chst4/Chst7* triple-deficient (GlcNAc6ST1,2,4 TKO) [11] mice were co-stained with CL40 (red) and anti-mesothelin (green) followed by Hoechst 33342 nuclear staining (blue). Both triple knockout mice showed negligible levels of CL40 signals in the mesothelium (open arrowheads). Digital images were captured using the same settings for each staining. The plot profiles of CL40 and mesothelin staining are shown (right). The signal intensities along the path of the line marker (dashed white line) in the merged images were measured, as described in Materials and Methods, with *n* = 3 for each genotype. (**B**) The relative intensity of CL40 to mesothelin is shown, with *n* = 12 mesothelium per genotype. Data are from three experiments with 4 pleural mesothelium analyzed in lung specimens from three donors for each genotype. **** *p* < 0.0001. Scale bar: 20 µm.

There may also be another possibility, that GlcNAc6ST2 is involved in the synthesis of 6-sulfo sLe^X^/sialyl 6-sulfo LacNAc, depending on the sulfation of intrinsic glycans by GlcNAc6ST3 and vice versa. Certain Golgi-resident carbohydrate sulfotransferases form complexes with other Golgi-resident enzymes [27]. GlcNAc6ST2 and GlcNAc6ST3 may form a heterodimer essential for the synthesis of CL40-reactive glycans in the pleural mesothelium. The activity of GlcNAc6ST3 depends on the presence of the core 2 structure in vitro [28] and in cultured cells [29]. GlcNAc6ST3 is not capable of synthesizing 6-sulfo sLe^X^ [29]. Therefore, the results of the present study and previous reports strongly support that CL40-reactive glycans in the pleura are sialyl 6-sulfo LacNAc existing within the core 2 branched structure of the *O*-linked glycans. Since GlcNAc6ST3 is also involved in the synthesis of R-10G-positive keratan sulfate [24], in which the predicted minimum epitope is a di-repeat of asialo 6-sulfo LacNAc [30], the possibility that the R-10G-reactive 6-sulfo LacNAc-repeated structure is also present in the pleural mesothelium is a future focus. We noted that the mesothelin-positive mesothelium was thickened in GlcNAc6ST2 KO mice and GlcNAc6ST3 KO mice. The mesothelial-cell layer may have been distorted for unknown reasons in these KO mice. We then generated mice triple-deficient in GlcNAc6ST1, 3, and 4, but sufficient in GlcNAc6ST2, and mice triple-deficient in GlcNAc6ST1, 2, and 4, but sufficient in GlcNAc6ST3. Interestingly, mesothelial CL40 immunoreactivity was negligible in both mice (Figure 4), indicating that neither GlcNAc6ST2 nor GlcNAc6ST3 was sufficient for the synthesis of pleural CL40 glycans. This is the first study to show that GlcNAc6ST2 and GlcNAc6ST3 complements mediate the synthesis of CL40-reactive sialylated and sulfated glycans in vivo. Specific deletion of the target gene in each KO lung was corroborated by real-time quantitative PCR (Figure S3). Thus, GlcNAc6ST2 and GlcNAc6ST3 are the sulfotransferases involved in the synthesis of the CL40 epitope in lung mesothelium, being different from lymph-node HEV where GlcNAc6ST2 and GlcNAc6ST1 are the sulfotransferases.

Siglecs (sialic acid-binding immunoglobulin-like lectins) recognize sialyl glycans and mediate cell signaling in the immune system [31,32,33,34]. Mouse Siglec-F recognizes 6′-sulfo sLe^X^ /sialyl 6′-sulfo LacNAc and sialylated non-sulfated bisected *N*-glycans. It was found that 6-sulfo sLe^X^/sialyl 6-sulfo LacNAc, but not 6′-sulfo sLe^X^/sialyl 6′-sulfo LacNAc, are present in mouse tracheal epithelial-cell mucins and bronchoalveolar-lavage (BAL) fluid [35,36,37]. Muc5b and Muc4, with molecular sizes of 200 kDa and >500 kDa, have been proposed to be the core proteins. Based on their glycan analysis, it was suggested that the major Siglec-F ligands are non-sulfated bisected *N*-glycans [35,36]. Synthetic enzymes and biological functions, if any, of 6-sulfo sLe^X^/sialyl 6-sulfo LacNAc in mouse tracheal epithelial mucins and BAL fluid will be the subject of future investigation alongside our current findings of CL40-reactive sialyl 6-sulfo glycans in the mesothelium. Human Siglec-8, a paralog of mouse Siglec-F, recognizes 6′-sulfo sLe^X^/sialyl 6′-sulfo LacNAc as well [38,39,40,41]. In humans, Siglec-8 ligands have been identified as sialyl keratan-sulfate aggrecan and DMBT1 [42,43]. These differences between species in Siglec-recognition and the core proteins of ligands are important issues to be considered. Care should be taken regarding these species-specific characteristics in future studies. Human Siglec-9 recognizes 6-sulfo sLe^X^/sialyl 6-sulfo LacNAc coated on a glycan array [44]. Whether or not the human-lung mesothelium generates CL40-reactive glycans, and if so, whether the glycans can be recognized by Siglec-9, is of great interest.

Currently, the physiological roles of mesothelial CL40-positive sialylated and sulfated glycans remain unknown. The mesothelium is a dynamic cellular membrane with many important functions, including the transport and movement of fluid and exogenous particulate matter across the serosal cavities, leukocyte migration during pathogen infection, and synthesis of pro-inflammatory cytokines, growth factors, and extracellular matrix proteins for tissue repair [45,46]. CL40 was first established as an antibody against HEV-specific sialomucins and as a functional agent that inhibits lymphocyte migration [8]. Given that CL40 blocks specific interactions between HEV sialomucins and lymphocyte L-selectin, our results suggest functional overlapping of CL40-positive 6-sulfo sLe^X^-glycans in HEVs and the pleural mesothelium, such as leukocyte migration. Possible inhibitory effects of CL40 on leukocyte–mesothelium interaction in lung pathology is of great interest to us. Mesothelial cells show epithelial phenotypes, including epithelial–mesenchymal transition (EMT), in response to transforming growth factor-ß [47,48]. Elucidating the potential role of CL40-positive glycans in the EMT of mesothelial cells may provide clues for understanding the physiological function of mesothelial CL40 glycans. Pleural mesothelial cells can migrate into the lung parenchyma and adopt a myofibroblast phenotype in idiopathic pulmonary fibrosis (IPF) [49,50]. The potential involvement of CL40-positive glycans in the parenchymal trafficking of mesothelial cells is a topic for future research. Further investigation of CL40 epitope expression in the pathological sets of mesothelioma and IPF is required.

The core proteins of the mesothelial CL40-positive glycans are unknown. Several sialomucins are expressed in the mesothelium [51,52]. CL40-positive high molecular mass glycoproteins modified with *O*-linked glycans may be included in these mesothelial sialomucins. One of them, MUC16, whose shed form is known as cancer antigen 125 (CA125), facilitates tumor-cell aggregation and its subsequent binding to the peritoneal surface via interaction with mesothelin in cancer cells [53,54]. Serum MUC16/CA125 levels are elevated in patients with malignant peritoneal mesothelioma [55]. Immune cells that do not express mesothelin have been shown to utilize Siglec-9 as an immune-cell receptor for MUC16 [56]. As described above, Siglec-9 can recognize the CL40-reactive glycan structure. We speculate that MUC16 could be a candidate core protein of mesothelial CL40-reactive glycans. The interaction between MUC16 in cancer cells and mesothelial cells mediates cancer-cell attachment to the mesothelium, which is the initial step of peritoneal dissemination in ovarian cancer [57,58,59]. Elucidating the role of CL40-reactive glycans in peritoneal or pleural metastasis of malignant cancer is an important challenge. The possible involvement of sulfation modification by GlcNAc6ST2 and GlcNAc6ST3 in the shedding of the transmembrane form of CL40 glycan core proteins is another topic for future research.

Notably, Siglec-9-binding to a cultured cell line was markedly enhanced by overexpression of human GlcNAc6ST2/CHST4 and GlcNAc6ST1/CHST2 [60], while the current results show a complementary action of GlcNAc6ST2 and GlcNAc6ST3 in mesothelial CL40 glycan. A previous study identified *CHST4* as a mesothelium-enriched gene [61], which strongly supports the involvement of GlcNAc6ST2 in the synthesis of the CL40 epitope in the mesothelium. In the human lungs, the contribution of GlcNAc6ST5/CHST6 to the synthesis of mesothelial CL40-reactive glycans is unknown [62]. This point needs to be clarified in future studies. The cell-type-specific regulation of CL40-reactive glycans is an important experimental approach. The generation of GlcNAc6ST2 and GlcnAc6ST3 doubly deficient (DKO) mice is necessary. Crossing each KO to obtain GlcNAc6ST2,3 DKO mice seems to be difficult because the chromosomal loci of *Chst4* and *Chst5* are close to each other in chromosome 8 [63]. A different approach to generate GlcNAc6ST2,3 DKO mice is required in future studies.

## 3. Materials and Methods

### 3.1. Antibodies and Enzymes

The CL40 monoclonal antibody was originally generated by immunizing GlcNAc6ST1,2 double-deficient mice with a 6-sulfo sLe^X^-terminated oligosaccharide [8]. CL40 is a murine IgG_1_ that recognizes 6-sulfo sLe^X^ and sialyl 6-sulfo LacNAc, as described previously [8]. The following materials were obtained commercially from the indicated sources. Rabbit polyclonal anti-mouse mesothelin antibody was from IBL (28127, Fujioka, Japan); Cy™ 3-conjugated goat anti-mouse IgG_1_ and Alexa Fluor 488-conjugated goat anti-rabbit IgG (H+L) were obtained from Jackson ImmunoResearch Laboratories (West Grove, PA); Endo-ß-galactosidase (*Escherichia freundii*) was from Seikagaku Corporation (Tokyo, Japan); α2-3,6,8 neuraminidase (*Arthrobacter ureafaciens*) was from Nacalai Tesque (Kyoto, Japan); and α1-3,4-fucosidase (*Streptomyces* sp. 142) was from Takara Bio Inc. (Shiga, Japan). Enzyme pretreatments were optimized previously [8,64].

### 3.2. Mice

GlcNAc6ST1-deficient (KO) (*Chst2*^−/−^) [3,22], GlcNAc6ST2-KO (*Chst4*^−/−^) [26], GlcNAc6ST3-KO (*Chst5*^−/−^) [23], and GlcNAc6ST4-KO (*Chst7*^−/−^) mice [24] were maintained on a C57BL/6J genetic background. GlcNAc6ST1,3,4 triple-deficient (TKO) mice were generated by crossbreeding GlcNAc6ST1,3 double-deficient and GlcNAc6ST4 KO mice [24]. GlcNAc6ST1,2,4 TKO mice were generated as described previously [11]. Male and female mice of all genotypes at 2 to 4 months old were used for the experiments. All mice were maintained under controlled specific-pathogen-free environmental conditions and provided with standard nourishment and water in the animal facilities of the authors’ institution.

### 3.3. Mouse Tissues

Mice were anesthetized and transcardially perfused with phosphate-buffered saline (PBS). The lung lobes and tracheas were dissected. Phosphate buffer (PB) containing 4% paraformaldehyde was infused through the airways. The left-lung lobe for frozen sectioning was post fixed overnight in PB containing 4% paraformaldehyde, equilibrated in 30% sucrose in PBS, and embedded in Tissue-Tek^®^ (O.C.T. compounds; Sakura, Torrance, CA, USA).

### 3.4. Immunohistochemistry and Fluorescence Microscopy

Frozen lung tissues were cut into 10-µm-thick sections via a cryostat and collected on MAS-coated glass slides (SF17293; Matsunami, Osaka, Japan). Sections were air-dried for 30 min, rinsed with PBS to remove O.C.T. compounds, and blocked in PBS containing 5% normal goat serum (Vector Laboratories, Burlingame, CA, USA) and 0.1% Triton-X 100 for 1 h at room temperature. For pretreatment, the sections were digested with α2-3,6,8 neuraminidase (200 mU/mL), endo-ß-galactosidase (10 mU/mL), or α1-3,4 fucosidase (0.05 mU/mL) in 50 mM Tris-acetate buffer pH 7.0 at 37 °C for 24 h. The sections were incubated with CL40 (10 µg/mL) and anti-mesothelin (1:200 dilution) in PBS containing 0.1% Triton-X 100 at 4 °C overnight. Sections were washed with PBS and incubated with a mixture of Cy™ 3-anti-mouse IgG_1_ (1:250 dilution) and Alexa Fluor 488-conjugated goat anti-rabbit IgG (H+L) (1:250 dilution) for 30 min at room temperature. After washing with PBS, the sections were counterstained with Hoechst to visualize the nuclei. The stained sections were mounted using FluorSave™ Reagent (Merck, Darmstadt, Germany). Signals were visualized and captured using a fluorescence microscope (BX41 microscope; Olympus, Tokyo, Japan) at the same exposure settings for each antibody. The fluorescence intensities of Cy™ 3-CL40 and Alexa Fluor 488-mesothelin in stained pleura of digital images were determined semiquantitatively by ImageJ (NIH, Bethesda, MD, USA; https://imagej.nih.gov/ij/ (accessed on 18 June 2018). Four pleural mesothelium per mouse were randomly selected. Three mice for each genotype or treatment were tested.

### 3.5. Statistical Analysis

All data are presented as means ± SE unless noted otherwise. The values were analyzed by one-way ANOVA with Dunnett’s test (vs. WT or no enzyme control) with Prism software (GraphPad Software, La Jolla, CA, USA). *p*-values less than 0.05 were considered to be statistically significant.

## Data Availability

The raw data supporting the conclusions of this article will be made available by the authors without undue reservation.

## Supplementary Materials

The following supporting information can be downloaded at: https://www.mdpi.com/article/10.3390/molecules27144543/s1, Figure S1: Immunohistochemical analysis of the mouse lung with control IgG1 and MECA-79; Figure S2: Immunoblotting analysis of the CL40 epitope in the mouse lung; Figure S3: mRNA expression levels of four members of the GlcNAc6ST family in the mouse lung of WT, GlcNAc6ST KOs and TKOs.

## Sample Availability

Samples generated in this study are available from the authors.
