# Supplementary material for: Complementary Role of GlcNAc6ST2 and GlcNAc6ST3 in Synthesis of CL40-Reactive Sialylated and Sulfated Glycans in the Mouse Pleural Mesothelium
Source: Molecules. 2022 Jul 16;27(14):4543. doi: 10.3390/molecules27144543 (PMC9320226; doi:10.3390/molecules27144543)
Supplement: Supplementary file 1 [file molecules-27-04543-s001.zip › molecules-1658662-supplementary.pdf]

Supplementary Materials

Complementary Role of GlcNAc6ST2 and GlcNAc6ST3 in Synthesis of CL40-Reactive Sialylated and Sulfated Glycans in the Mouse Pleural Mesothelium

Yoshiko Takeda-Uchimura[1], Midori Ikezaki[2], Tomoya O. Akama[3], Kaho Nishioka[4], Yoshito Ihara[2], Fabrice Allain[1], Kazuchika Nishitsuji[2] and Kenji Uchimura[1]*

[1]Univ. Lille, CNRS, UMR 8576 - UGSF - Unité de Glycobiologie Structurale et Fonctionnelle, Lille, France; yoshiko.uchimura@univ-lille.fr (Y.T.-U.); fabrice.allain@univ-lille.fr (F.A.); kenji.uchimura@univ-lille.fr (K.U.)
[2]Department of Biochemistry, School of Medicine, Wakayama Medical University, Wakayama 641-8509, Japan; nishit@wakayama-med.ac.jp (K.Nishitsuji); ikezaki@wakayama-med.ac.jp (M.I.); y-ihara@wakayama-med.ac.jp (Y.I.)
[3]Department of Pharmacology, Kansai Medical University, Osaka 570-8506, Japan; akamat@hirakata.kmu.ac.jp (T.-O.A.)
[4]Department of Obstetrics and Gynecology, School of Medicine, Wakayama Medical University, Wakayama 641-8509, Japan; ka-matsu@wakayama-med.ac.jp (K.Nishioka)

*Correspondence: kenji.uchimura@univ-lille.fr ; +33(0) 20 33 72 39

Figure S1

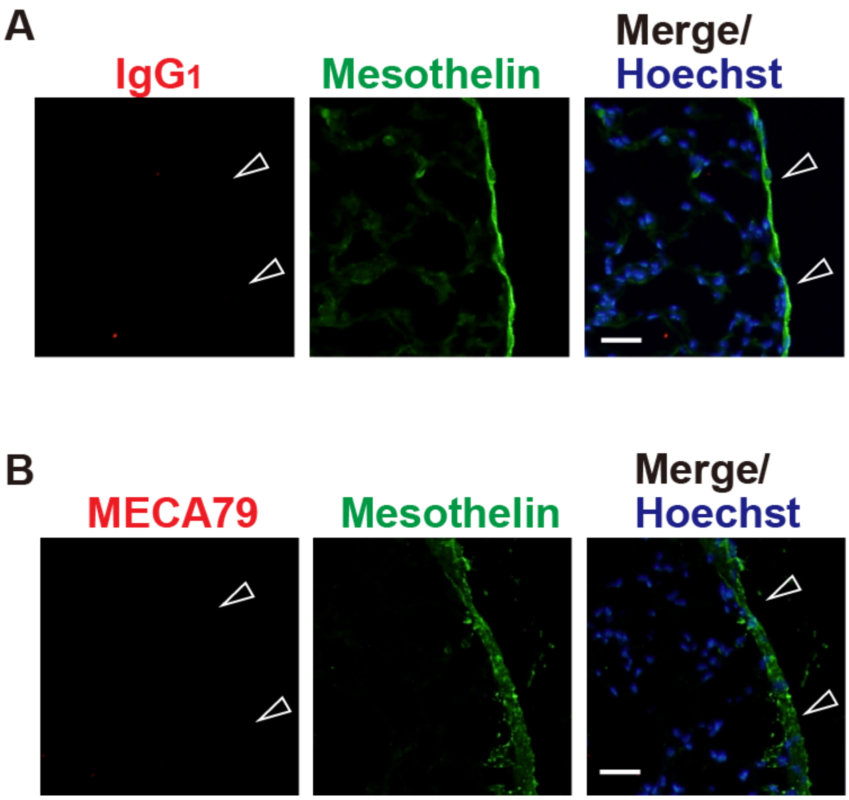

Figure S1. Immunohistochemical analysis of the mouse lung with control IgG1 and MECA-79.

Lung sections from normal adult mice were co-stained with control IgG1 (*red*) **A**, or MECA-79 (*red*) **B**, and an anti-mesothelin antibody (*green*) followed by Hoechst 33342 nuclear staining (*blue*). Representative fluorescence microscope images of the lower/middle region of left lung lobe are shown (n = 2). Staining signals with control IgG1 or MECA-79 in the pleural mesothelium are negligible (*open arrowheads*) revealed by co-stained signals with a mesothelium marker, mesothelin. Digital images were captured at the same setting for each staining. Scale bar: 20 µm. Control IgG1 was negative in the upper region of lung lobes where CL40 also showed the intense mesothelial signals (not shown).

Figure S2

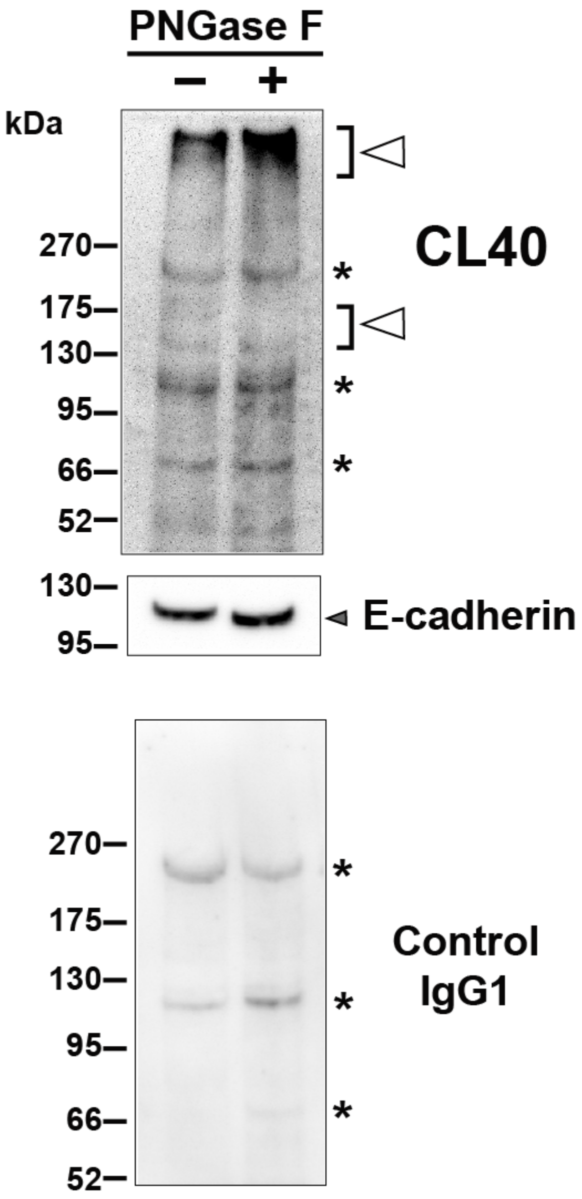

Figure S2. Immunoblotting analysis of the CL40 epitope in the mouse lung.

One % Triton X 100-soluble fractions were prepared from tissue homogenates of 100 mg lung lobes of 2- to 3-month old C57BL6 mice. The fractions were incubated with GlcNAc-binding wheat germ agglutinin (WGA)-coated beads (Vector Laboratories, Burlingame, CA) at 4 °C overnight. Bead-bound materials were pretreated without or with PNGase F (New England Biolabs, Ipswich, MA). Immunoblot with CL40 or isotype-match control IgG1 was performed as described previously (Arata-Kawai H et al, *Am J Pathol* 2011, 178:423–433; doi:10.1016/j.ajpath.2010.11.009). Bands with molecular weights of > 270 kDa and 145-175 kDa indicated by open arrowheads were observed. Bands with 70 kDa, 120 kDa, and 240 kDa indicated by asterisks were also seen in IgG1 control blots. E-cadherin was used to show protein equal loading and successful pretreatment of PNGase F on the lung fraction. Closed arrowhead indicates the 110 kDa band shifted from 120 kDa in the pretreated fraction. Representative results are shown (n = 2).

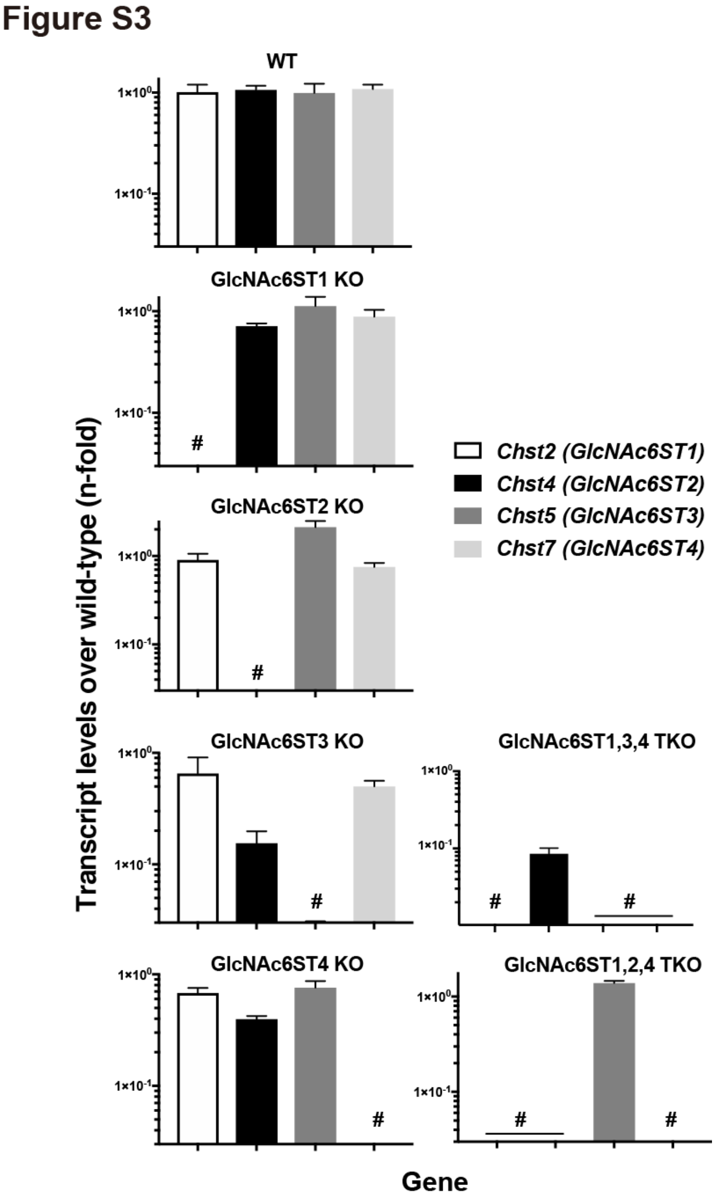

Figure S3. mRNA expression levels of four members of the GlcNAc6ST family in the mouse lung of WT, GlcNAc6ST KOs and TKOs.

Total RNA from the lung lobes of adult WT, GlcNAc6ST1 KO, GlcNAc6ST2 KO, GlcNAc6ST3 KO, GlcNAc6ST4 KO, GlcNAc6ST1,3,4 triple deficient (TKO), and GlcNAc6ST1,2,4 TKO mice were prepared and tested. Transcript levels of *Chst2* (GlcNAc6ST1), *Chst4* (GlcNAc6ST2), *Chst5* (GlcNAc6ST3), and *Chst7* (GlcNAc6ST4) were determined by real-time quantitative PCR as described previously (Zang Z et al, *Proc Natl Acad Sci USA* 2017, 114, E2947-E2954; doi:10.1073/pnas.1615036114). The primers for *Chst2*, *Chst5*, and *Chst7* were used as described previously (Zang Z et al, *Proc Natl Acad Sci USA* 2017, 114, E2947-E2954; doi:10.1073/pnas.1615036114). The primer sequences for *Chst4* were as follows: 5′- CGGATGTGTTCTACCTGATGGAG -3′ (forward) and 5′- CACAGGAAGACGGAACGCAGAA -3′ (reverse). Data are presented as means ± SD. #, data below the limit of detection.
